# Metallosis-Induced Warm Antibody Auto-Immune Hemolytic Anemia After Bilateral, Large-Diameter Metal-on-Metal Total Hip Arthroplasty With Complete Remission After Revision

**DOI:** 10.1016/j.artd.2024.101471

**Published:** 2024-08-03

**Authors:** Alexander Frank Heimann, Emanuel Gautier, Joseph M. Schwab, Peter Wahl, Moritz Tannast, Emmanuel Levrat, Ines Raabe

**Affiliations:** a Department of Orthopaedic Surgery and Traumatology, HFR Fribourg – Cantonal Hospital, University of Fribourg, Fribourg, Switzerland; b Division of Orthopaedics and Traumatology, Cantonal Hospital Winterthur, Winterthur, Switzerland; c Faculty of Medicine, University of Berne, Berne, Switzerland; d Department of Hematology, HFR Fribourg – Cantonal Hospital, University of Fribourg, Fribourg, Switzerland

**Keywords:** Hemolytic anemia, Metal-on-metal, Total hip arthroplasty, Wear, Complete remission, LH-MoM THA

## Abstract

The use of metal-on-metal bearing couples in total hip arthroplasty can lead to an increased release of metal ions, particularly cobalt and chromium over time. This can lead to local and systemic metallosis, which has cytotoxic, genotoxic, and immunotoxic effects and can cause a host of secondary disorders. We describe the case of a 37-year-old female patient that was diagnosed with warm-antibody autoimmune hemolytic anemia (WAIHA) one and a half years after bilateral large-diameter head metal-on-metal total hip arthroplasty. For 11 years, it was refractory to all therapy, including splenectomy and rituximab, requiring long-term oral prednisone for disease control. Ultimately, systemic metallosis and periprosthetic joint infection were diagnosed, requiring explantation of the prostheses. By the sixth week postoperatively, she experienced complete spontaneous remission of her WAIHA. In conclusion, WAIHA can be associated with systemic metallosis in patients with metal-on-metal prosthetic joint replacements. Both hematologists and orthopedic surgeons should be aware of this.

## Introduction

Autoimmune hemolytic anemia (AIHA) is a hematological disorder mainly characterized by autoantibody-mediated red blood cell depletion [1]. It has a prevalence of 9.5 per 100,000 people [2]. Those affected by this relatively rare disease suffer from predominantly extravascular hemolysis with varying degrees of clinical symptoms, ranging from fatigue, pallor, and weakness to life-threatening hemolytic crises. Depending on the temperature at which the antigen-antibody reactions primarily occur, a distinction is made between cold AIHA and warm AIHA (WAIHA). Although various causes of WAIHA have been identified in the past, including other autoimmune diseases, cancers, or viral infections [3], most cases are still considered idiopathic.

In joint replacement, the quest for durable materials has led to the use of metal-on-metal (MoM) bearing couples in total hip arthroplasty (THA). Despite the initial widespread use, concerns about possible complications associated with MoM implants have increased over time. The release of metal wear particles and ions into the body, resulting in the secondary development of local and systemic complications, has been particularly associated with this bearing [4]. To the best of our knowledge, there are 2 reports in the literature that link systemic metal toxicity with hematologic disorders [5,6]. However, a possible link between systemic metal toxicity after MoM-THA and autoimmune phenomena such as WAIHA has not yet been reported. Therefore, we report a case of WAIHA, refractory to usual treatment modalities, whose onset and remission was coincident with the implantation and removal of bilateral large-diameter MoM THA, complicated by local and systemic metal wear particle disease.

## Case history

The patient described in this report gave her written informed consent for publication of anonymized data. A 36-year-old, otherwise healthy, smoking female (20 pack years) with a body mass index of 25 kg^.^m^2^ underwent within a month primary staged bilateral THA via a posterolateral approach for secondary osteoarthritis due to developmental dysplasia [7]. Both THA had MoM implants utilizing a monoblock metal acetabular cup (Durom, Zimmer Biomet, Warsaw, IN), a large-diameter metal head (40 mm on the right and 38 mm on the left, Metasul LDH, Zimmer Biomet) with the appropriate sleeve adapter, and a cementless femoral stem (CLS Spotorno, Zimmer Biomet). The early postoperative course was without complications, and the patient reported being pain free at 3-month follow-up.

### Initial clinical presentation

Eighteen months after surgery, the patient presented fatigue, dyspnea, and jaundice. Clinical examination revealed a normotensive patient with a normal resting heart rate. No heart murmurs or organomegaly were detected. Laboratory analysis revealed hyperchromic, macrocytic anemia with a hemoglobin of 70 g/l, normal white blood count (4.7 G/l), and slightly elevated platelet count (435 G/l). She received intramuscular vitamin B12 and intravenous iron substitution from her general practitioner.

### Hematologic workup

At 1-week follow-up, hemoglobin concentration further decreased to 63 g/l, and reticulocyte count increased to 397/1000 (norm 6-22/1000). The patient was then admitted to our hematology service, and further workup, including blood smear and bone marrow aspiration, was performed. Hyperchromic, macrocytic anemia with hemolytic signs (Table 1) were confirmed. Direct Coombs test (antiglobulin test) was positive with an IgG titer of 1:32 [8,9]. Blood smear showed pronounced anisocytosis, clear polychromasia, and agglutinate formation. Granulocytes were normally segmented, with fine granulation. Thrombocytes also showed anisocytosis. Bone marrow aspiration revealed hypercellular hematopoietic bone marrow with considerable hyperplasia of the erythroid series, slight hypoplasia of the granulocyte series, and mild lymphoplasmocytosis, interpreted as reactive. These findings led to the diagnosis of idiopathic WAIHA. Treatment with oral prednisone was initiated.

**Table 1:** Evolution of relevant blood values over time.

Parameter	Normal range	Initial diagnosis	Before revision surgery	At 8 weeks postoperatively	At 7 months postoperatively	At 1 year postoperatively	Last follow-up at 6.5 years postoperatively
Hemoglobin	120-160 g/l	62 g/l	84 g/l	138 g/l	148 g/l	128 g/l	142 g/l
Erythrocytes	4.00-5.00 T/l	1.54 T/l	2.65 T/l	4.50 T/l	4.92 T/l	4.65 T/l	4.218 T/l
Leukocytes	4.0-10.0 G/l	4.7 G/l	16.9 G/l	5.1 G/l	6.5 G/l	9.4 G/l	5.2 G/l
Thrombocytes	150-370 G/l	435 G/l	1480 G/l	438 G/l	435 G/l	504 G/l	369 G/l
MCH	29-35 pg	40 pg	32 pg	31 pg	30 pg	28 pg	34 pg
MCV	80-98 fl	117 fl	100 fl	91 fl	92 fl	87 fl	99 fl
MCHC	320-370 g/l	344 g/l	318 g/l	336 g/l	329 g/l	318 g/l	345 g/l
Reticulocytes	6-22/1000	370/1000	99/1000	15/1000	17/1000	18/1000	29/1000
Ferritin	30-150 μg/l	134 μg/l	1244 μg/l	-	156 μg/l	-	212 μg/l
LDH	<450 U/l	1196 U/l	938 U/l	271 U/l	275 U/l	316 U/l	278 U/l
Total bilirubin	3.1-18.8 μmol/l	47.5 μmol/l	34.5 μmol/l	3.3 μmol/l	-	-	8.1 μmol/l
Direct bilirubin	<3.4 μmol/l	7,0 μmol/l	21.6 μmol/l	2.2 μmol/l	-	-	3.3 μmol/l
Haptoglobin	0.35-2.0 g/l	-	<0.10 g/l	0.75 g/l	0.78 g/l	1.08 g/l	1.38 g/l

LDH, lactate dehydrogenase; MCH, mean corpuscular hemoglobin; MCV, mean corpuscular volume; MCHC, mean corpuscular hemoglobin concentration.

### Mid-term to long-term evolution

Persistent hemolytic activity was observed throughout the following year, even under continued steroid therapy. Computed tomography (CT) scan of the thorax and abdomen revealed a normal spleen, pancreas, and adrenal glands and revealed no tumor. After 13 months, an unsuccessful discontinuation of the cortisone therapy was attempted. Laparoscopic splenectomy was subsequently performed 2 months later. However, hemolysis persisted despite continued immunosuppression with prednisone. Two years later, nearly 5 years after THA, the patient developed bilateral, segmental, and subsegmental pulmonary embolism, prompting anticoagulation with a vitamin K antagonist (acenocoumarol). Thrombophilia workup (Factor V Leiden mutation, prothrombin 20,210 mutation, protein C, protein S, antithrombin, factor VIII, autoantibody diagnostics for antiphospholipid syndrome [lupus anticoagulant, cardiolipin antibodies, β2-glycoprotein I antibodies]) did not show any particularities. A further year later, a trial with 4 cycles of rituximab (500 mg) was initiated. While this allowed a reduction of prednisone to 7.5 mg/day within a year, further reduced to 5 mg/day another year later, there was persistent hemolytic activity. Meanwhile, the patient contracted Lyme arthritis, treated with 3 weeks of intravenous ceftriaxone, which temporarily worsened her WAIHA.

**Figure 1:**
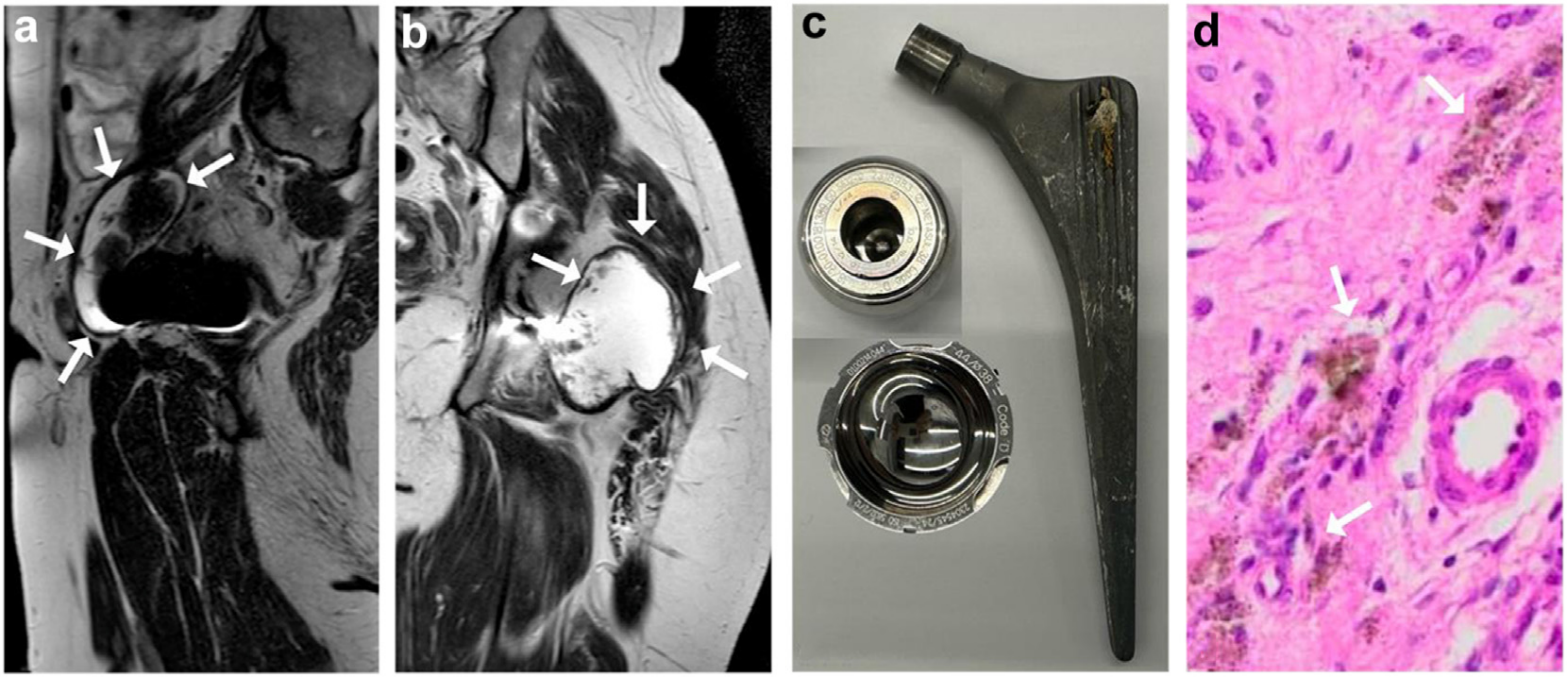
Sagittal (a) and coronal (b) T2 metal artifact reduction sequence (MARS) MR images showing a prominent fluid collection along the iliopsoas tendon with a hypointense margin and heterogeneous appearance on the right (a) and a large collection with hypointense border and heterogeneous appearance on the left (b), characteristic of an adverse reaction to metal debris (white arrows). Picture of the explanted left, large-head, metal-on-metal total hip arthroplasty (c). Histologic image (d) of the excised pseudotumor with deposits of metal debris and pigmented macrophages (white arrows).

After 11 years postoperatively, the patient consulted her surgeon as she started complaining of intermittent bilateral hip pain. She had elevated serum cobalt (22.3 μg/1, norm: <1.3) and chromium (3.6 μg/1, norm: <1.1) levels at that time (Table 2). An MRI of the pelvis revealed a small posterior collection extending from the right hip joint and a larger collection along the right iliopsoas tendon (maximal craniocaudal extension of 70 mm, Fig. 1a). On the left side, it revealed a voluminous posterior collection (maximum diameter of 80 mm, Fig. 1b) extending from the hip joint and a small collection anterior to the anterior acetabular wall. These collections were consistent with adverse reaction to metal debris (ARMD) [3]. These findings led to the diagnosis of local and systemic metallosis. The treating orthopedic surgeon recommended observation. No further infection-related diagnostic tests were carried out at this time.

**Table 2:** Evolution of serum levels of cobalt and chromium over time.

Metal ion	Normal range	Before revision	3 days	6 weeks	12 weeks	6 months	3.5 years	Last follow-up 6.5 years
Cobalt	<1.3 μg/1	22.3	*4.4*	*6.2*	**0.6**	**0.6**	**1.1**	**1.1**
	<20 nmol/l	378	75	105	11	11	18	18
Chromium	<1.1 μg/1	3.6	3.9	1.9	2.6	2.3	0.8	0.5
	<20 nmol/l	69	75	36	51	45	17	11

Values below the reported threshold for clinical concern (SCENIHR Final Opinion 2014) for cobalt of 2 to 7 μg/1 are given in italic. Values in the normal range are in bold.

**Figure 2:**
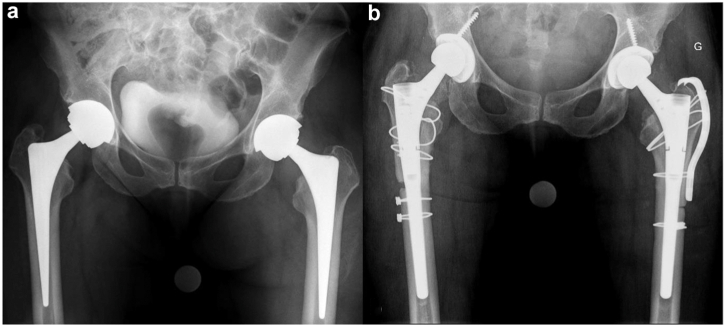
Anteroposterior pelvis radiograph (a) prior to and (b) 5 months after bilateral revision for systemic metal toxicity and bilateral periprosthetic joint infection.

Two months later, the patient had to be hospitalized emergently because of a septic shock. Repeated imaging revealed increased fluid accumulation in both the left and right psoas muscles. *Staphylococcus aureus* was identified in blood cultures, in computed tomography–guided aspiration of the fluid collections, as well as in aspirations of both hip joints. Targeted intravenous antibiotics were administered, and the oral anticoagulation reversed. A bilateral, 2-stage revision using antibiotic-loaded spacers of both THA with drainage of the psoas abscesses was performed. Both THA were explanted sequentially within days (Fig. 1c). Histologic examination of surgical tissue specimens showed signs of infection and metallosis (Fig. 1d). After completion of the antibiotic therapy, reimplantation of bilateral THA with a ceramic-on-highly cross-linked polyethylene bearing was performed (Fig. 2).

### Spontaneous complete remission of the WAIHA

**Figure 3:**
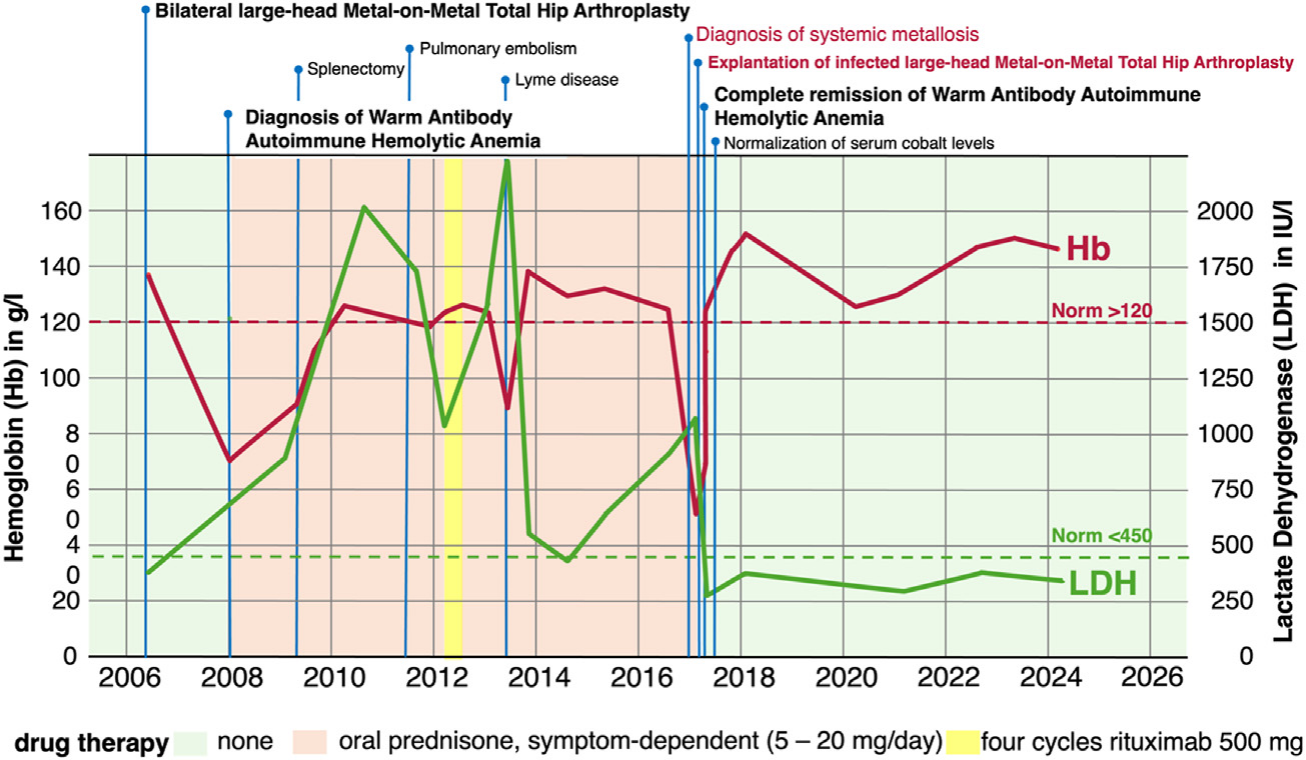
Schematic representation of the course of the serum hemoglobin (Hb) and lactate dehydrogenase (LDH) value over time depending on the most important events in the patient's history and associated drug therapy.

Six weeks after explantation, the patient showed spontaneous normalization of her intravascular hemolysis and hemoglobin levels. Based on this finding, cortisone therapy was slowly reduced and then completely discontinued, without recurrence of the WAIHA. Serum cobalt levels returned to normal at 12 weeks, whereas normalization of chromium was observed only later (Table 2). Since then, the patient is in complete remission and symptom-free without any treatment, after more than 5 years of follow-up (Fig. 3).

## Discussion

In the past, the use of MoM bearings was introduced to reduce wear in THA. Expected reduced wear led to the use of large-diameter heads, to improve range of motion and reduce dislocation rates. LH MoM were thus preferably implanted in young and active patients [10]. As a result, this bearing has been implanted in more than 100,000 patients in Europe alone [4]. However, this type of implant has an increased risk of metal ion release, specifically cobalt and chromium. The particles can originate either from the bearing itself or from the taper junction between the head and stem (trunnoniosis) [11]. This is particularly relevant for large metal heads, as these place a greater load on the taper junction [12]. Eventually, this wear particle and metal ion release can result in local ARMD and increased systemic metal ion concentrations [13]. In our case, there were macroscopic signs of metal wear at the taper junction (Fig. 1c), and the patient had an elevated cobalt/chromium ratio, further indicating that trunnoniosis was the most likely cause of systemic metal toxicity [14]. The bilateral ARMD were also consistent with this hypothesis [15].

Systemic exposure to metal ions, particularly cobalt and chromium, the metals used in MoM bearings, has cytotoxic, genotoxic, and immunotoxic effects and may cause a host of secondary disorders [[16], [17], [18]]. The cytotoxicity of metal debris strongly depends on the probability of intracellular uptake. While microparticles and nanoparticles typically enter cells by endocytosis or phagocytosis and depend on several factors such as size, surface charge, and shape, the valence of the metal ions plays a decisive role in the probability of uptake and consequently their cytotoxicity [19]. Based primarily on in-vitro studies, it is currently assumed that cobalt ions have a higher cytotoxic potential than chromium ions [20], and that a small particle size increases the cytotoxicity [21]. To date, there are no clinical data to support an increased incidence of cancer or increased mortality for patients with MoM implants [22]. However, the long-term effects of exposure to metal debris are still unknown. In vitro, cobalt and chromium have genotoxic properties, with chromium appearing to have a stronger genotoxic effect [16]. They inhibit DNA repair mechanisms, induce single- and double-strand breaks in DNA, and can cause simple or complex aneuploidy even at physiological doses, which has also been demonstrated in peripheral lymphocytes from patients with MoM implants [[23], [24], [25]]. The observed aberrations correlated with the metal-ion concentration, and the detectable damage was lower after a switch to metal-on-polyethylene bearing [26]. Immunotoxic effects of wear particles are thought to be a major contributor to the development of local ARMD. In the context of total joint replacement, a lymphocyte-dominated (CD8+), cell-mediated (type IV) hypersensitivity reaction has been postulated as the main mechanism, while genetic predisposition has also been discussed [27,28]. In addition, systemic immunotoxic reactions such as peripheral eosinophilia, cognitive impairment, and fatigue have recently been reported in patients with MoM implants [17,29]. A common thread in these secondary disorders is the near-complete symptom relief after explantation, typically accompanied by a reduction in serum chromium and cobalt levels [4,29].

Apart from this case, there are only 2 other case reports available in the literature establishing a link between arthroplasty-related metal toxicity and AIHA, but both differ significantly [5,6]. In the case described by Nakamura et al. [5], chromium levels were within the normal range and did not change postoperatively, nickel levels were only slightly elevated, but cobalt levels were not reported. Even though autoantibodies persisted after revision, the authors reported a resolution of the anemia, similarly to our study [5]. Duarte et al [6] described a case in which a broken ceramic inlay led to direct contact between a ceramic ball head and a titanium-aluminum-vanadium alloy cup, causing audible noise during normal gait. Two months after revision, the patient developed AIHA. Imaging revealed a large intrapelvic collection, similar to the one observed in our case, and after complete replacement of all prosthetic components and drainage of the abscess, the patient’s AIHA resolved [6].

Monitoring of systemic metal ion exposure after implantation of MoM bearings can be done in whole blood, plasma, and urine [30]. The choice of determination in whole blood or in serum is influenced by the laboratory techniques available. Technical aspects of the assay also influence results. Even if, in our case, only serum concentrations were available, determination in whole blood is recommended. Although there are no strict reference values or monitoring guidelines, a critical threshold range of 2-7 μg/l for cobalt in whole blood is recommended, with comorbidities possibly caused by metal toxicity to be considered [4,17,18]. Our patient’s preoperative blood levels were well above this threshold, particularly for cobalt. At 6 weeks postoperatively, the values had decreased to a range within the threshold and remained well below during the entire follow-up period (Table 2).

It is important to point out that the patient not only had elevated serum metal ion levels but also a periprosthetic joint infection, the onset of which is not entirely clear. As our treatment resolved both problems, and the patient subsequently showed complete remission of her WAIHA, we cannot say with absolute certainty which of the factors were causative. However, the normalization of the cobalt ion levels coinciding with complete spontaneous remission of WAIHA strongly suggests an association, particularly as both the acute clinical presentation and the isolated bacterium are more likely to be associated with an acute periprosthetic joint infection than a chronic low-grade infection.

## Summary

Considering the striking association between the resolution of WAIHA and systemic metal ion toxicity after revision, cobalt or chromium toxicity secondary to MoM THA must be postulated as being causative. Although rare, WAIHA represents a life-threatening disease. Both hematologists and orthopedic surgeons should be aware of this, and patients with MoM THA should be monitored regularly, including determination of cobalt and chromium levels in whole blood.

## Acknowledgment

This work was performed at the HFR Fribourg – Cantonal Hospital, Department of Orthopedic Surgery and Traumatology, Fribourg, Switzerland.

## Conflicts of interest

JMS is a paid consultant for DePuy Synthes (a Johnson & Johnson company) and is an unpaid consultant for Mizuho OSI. PW is a member (unpaid) in the expert group of the Swiss National Joint Registry and in the expert infection group of Swiss Orthopaedics. MT is a paid consultant for DePuy Synthes and Lima, receives research support from DePuy Synthes, and is a board member of Swiss Orthopaedics. All other authors declare no conflicts to disclose.

For full disclosure statements, refer to https://doi.org/10.1016/j.artd.2024.101471.

## Informed patient consent

The author(s) confirm that written informed consent has been obtained from the involved patient(s) or if appropriate from the parent, guardian, power of attorney of the involved patient(s); and, they have given approval for this information to be published in this case report (series).

## CRediT authorship contribution statement

**Alexander Frank Heimann:** Writing – review & editing, Writing – original draft, Investigation. **Emanuel Gautier:** Supervision, Data curation, Conceptualization. **Joseph M. Schwab:** Visualization, Formal analysis, Data curation. **Peter Wahl:** Writing – review & editing, Validation, Investigation. **Moritz Tannast:** Supervision, Methodology, Conceptualization. **Emmanuel Levrat:** Investigation, Formal analysis, Conceptualization. **Ines Raabe:** Writing – original draft, Supervision, Project administration, Conceptualization.

## Appendix A Supplementary Data

Conflict of Interest Statement for Heimann

Conflict of Interest Statement for Gautier

Conflict of Interest Statement for Levrat

Conflict of Interest Statement for Raabe

Conflict of Interest Statement for Schwab

Conflict of Interest Statement for Tannast

Conflict of Interest Statement for Levrat
