# Supplementary material for: Metallosis-Induced Warm Antibody Auto-Immune Hemolytic Anemia After Bilateral, Large-Diameter Metal-on-Metal Total Hip Arthroplasty With Complete Remission After Revision
Source: Arthroplast Today. 2024 Aug 3;29:101471. doi: 10.1016/j.artd.2024.101471 (PMC11342758; doi:10.1016/j.artd.2024.101471)
Supplement: Conflict of Interest Statement for Gautier [file mmc2.pdf]

CONFLICT OF INTEREST STATEMENT

American Association of Hip and Knee Surgeons

(Adopted from the American Academy of Orthopaedic Surgeons disclosure statement)

The following form **must be filled out completely and submitted by each author (example, 6 authors, 6 forms). All items require a response. If there is no relevant disclosure for a given item, enter "*None*."**

Manuscript Title
Metallosis-induced warm antibody auto-immune hemolytic anemia after bilateral large diameter metal-on-metal total hip replacement with complete remission after prosthesis replacement

1. Royalties from a company or supplier (The following conflicts were disclosed)
   No

2. Speakers bureau/paid presentations for a company or supplier (The following conflicts were disclosed)
   No

3A. Paid employee for a company or supplier (The following conflicts were disclosed)
   No

3B. Paid consultant for a company or supplier (The following conflicts were disclosed)
   No

3C. Unpaid consultants for a company or supplier (The following conflicts were disclosed)
   No

4. Stock or stock options in a company or supplier (The following conflicts were disclosed)
   No

5. Research support from a company or supplier as a Principal Investigator (The following conflicts were disclosed)
   No

6. Other financial or material support from a company or supplier (The following conflicts were disclosed)
   No

7. Royalties, financial or material support from publishers (The following conflicts were disclosed)
   No

8. Medical/Orthopaedic publications editorial/governing board (The following conflicts were disclosed)
   No

9. Board member/committee appointments for a society (The following conflicts were disclosed)
   No

Each author must sign AND print or type his/her name, date and submit a separate form

In addition, one BLINDED Conflict of Interest form (no author names used) should be submitted per manuscript with all author disclosures.

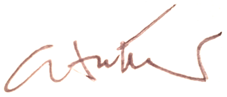

Emanuel GAUTIER		April 30, 2024
Author Name (Print or Type)	Author Signature	Date
