# Supplementary material for: Metallosis-Induced Warm Antibody Auto-Immune Hemolytic Anemia After Bilateral, Large-Diameter Metal-on-Metal Total Hip Arthroplasty With Complete Remission After Revision
Source: Arthroplast Today. 2024 Aug 3;29:101471. doi: 10.1016/j.artd.2024.101471 (PMC11342758; doi:10.1016/j.artd.2024.101471)
Supplement: Conflict of Interest Statement for Raabe [file mmc4.pdf]

CONFLICT OF INTEREST STATEMENT

American Association of Hip and Knee Surgeons

(Adopted from the American Academy of Orthopaedic Surgeons disclosure statement)

Metallosis-induced warm antibody auto-immune hemolytic anemia after bilateral large diameter metal-on-metal total hip replacement with complete remission after prosthesis replacement

---

1. Royalties from a company or supplier (The following conflicts were disclosed) **None**

2. Speakers bureau/paid presentations for a company or supplier (The following conflicts were disclosed) **None**

3A. Paid employee for a company or supplier (The following conflicts were disclosed) **None**

3B. Paid consultant for a company or supplier (The following conflicts were disclosed) **None**

3C. Unpaid consultants for a company or supplier (The following conflicts were disclosed) **None**

4. Stock or stock options in a company or supplier (The following conflicts were disclosed) **None**

5. Research support from a company or supplier as a Principal Investigator (The following conflicts were disclosed) **None**

6. Other financial or material support from a company or supplier (The following conflicts were disclosed) **None**

7. Royalties, financial or material support from publishers (The following conflicts were disclosed) **None**

8. Medical/Orthopaedic publications editorial/governing board (The following conflicts were disclosed) **None**

9. Board member/committee appointments for a society (The following conflicts were disclosed) **None**

Ines Raabe, MD1	[signature]	May 01, 2024
Author Name (Print or Type)	Author Signature	Date
