# Supplementary material for: Metallosis-Induced Warm Antibody Auto-Immune Hemolytic Anemia After Bilateral, Large-Diameter Metal-on-Metal Total Hip Arthroplasty With Complete Remission After Revision
Source: Arthroplast Today. 2024 Aug 3;29:101471. doi: 10.1016/j.artd.2024.101471 (PMC11342758; doi:10.1016/j.artd.2024.101471)
Supplement: Conflict of Interest Statement for Schwab [file mmc5.pdf]

CONFLICT OF INTEREST STATEMENT

American Association of Hip and Knee Surgeons

(Adopted from the American Academy of Orthopaedic Surgeons disclosure statement)

The following form **must be filled out completely and submitted by each author (example, 6 authors, 6 forms).**
All items require a response. If there is no relevant disclosure for a given item, enter "*None*."

Metallosis-induced warm antibody auto-immune hemolytic anemia after bilateral large diameter metal-on-metal total hip replacement with complete remission after prosthesis replacement

Manuscript Title

1. Royalties from a company or supplier (The following conflicts were disclosed) None

2. Speakers bureau/paid presentations for a company or supplier (The following conflicts were disclosed) None

3A. Paid employee for a company or supplier (The following conflicts were disclosed) None

3B. Paid consultant for a company or supplier (The following conflicts were disclosed)

DePuySynthes – Johnson & Johnson – paid consultant

3C. Unpaid consultants for a company or supplier (The following conflicts were disclosed)

Mizuho OSI – unpaid consultant

4. Stock or stock options in a company or supplier (The following conflicts were disclosed) None

5. Research support from a company or supplier as a Principal Investigator (The following conflicts were disclosed) None

6. Other financial or material support from a company or supplier (The following conflicts were disclosed) None

7. Royalties, financial or material support from publishers (The following conflicts were disclosed) None

8. Medical/Orthopaedic publications editorial/governing board (The following conflicts were disclosed) None

9. Board member/committee appointments for a society (The following conflicts were disclosed) None

Each author must sign AND print or type his/her name, date and submit a separate form

In addition, one BLINDED Conflict of Interest form (no author names used) should be submitted per manuscript with all author disclosures.

Joseph Schwab	[signature]	April 30, 2024
Author Name (Print or Type)	Author Signature	Date
